# Supplementary material for: Impact of alternative splicing on Arabidopsis proteome
Source: Nucleic Acids Res. 2026 May 4;54(8):gkag400. doi: 10.1093/nar/gkag400 (PMC13136898; doi:10.1093/nar/gkag400)
Supplement: gkag400_Supplemental_Files [file gkag400_supplemental_files.zip › Reyes_NAR_supplementalFigures202604.pdf]

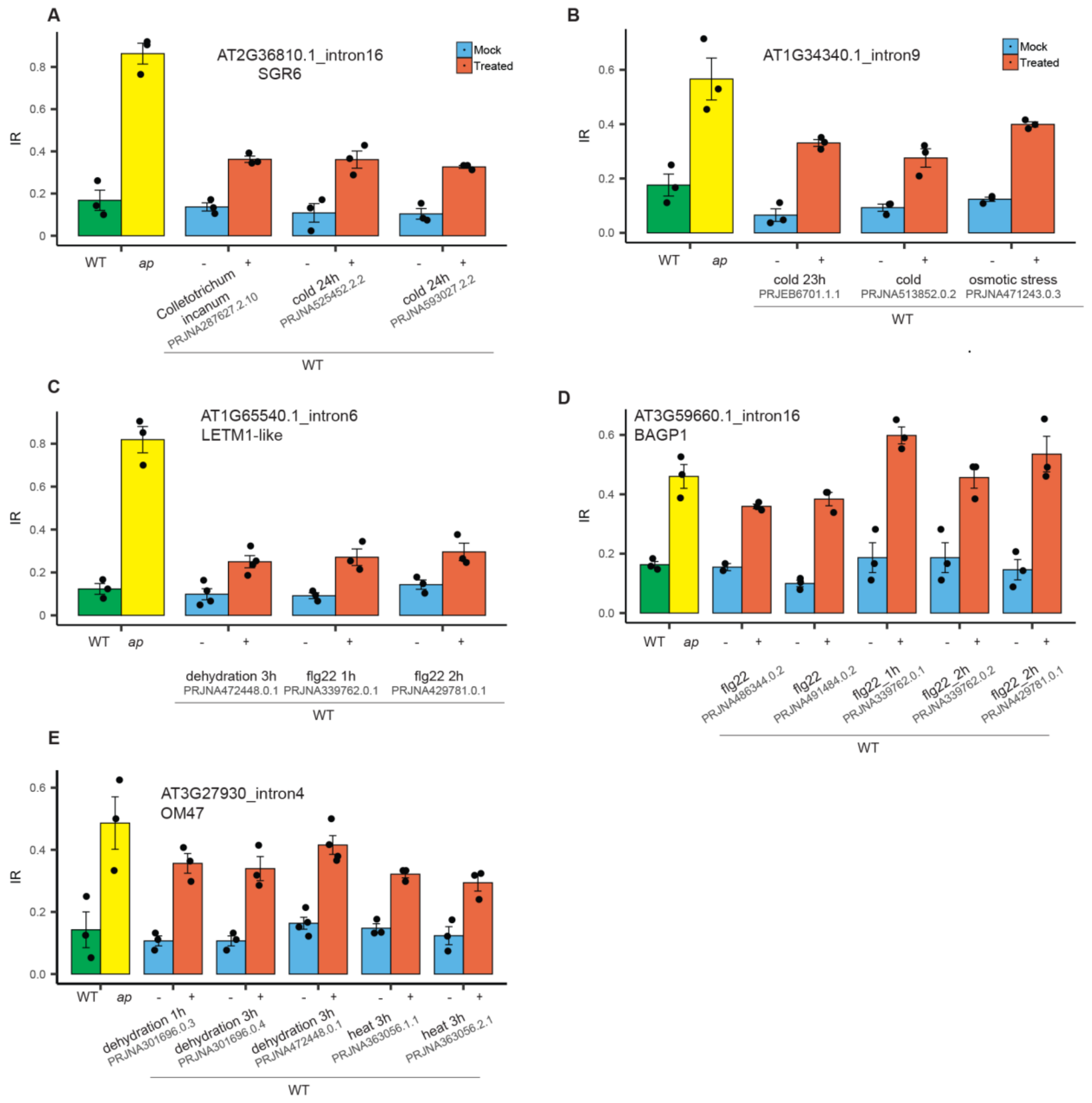

Supplementary Figure S1. Intron retention (IR) events observed in the *acinus pinin* mutants are physiologically recapitulated in wild-type plants under biotic and abiotic stress conditions, as analyzed using *PlantIntronDB*.

All IR events shown are significantly different from their respective controls (student t=test, $p \leq 0.05$).

(**A-E**) Bar graphs show five representative IR events altered in the *acinus pinin* mutant and also in wild-type plants following cold, osmotic, dehydration, flagellin, fungi, or heat treatment. The specific RNA-seq project names are indicated. Error bars represent the mean ± the standard deviation. The IR data for the *acinus pinin* (*ap*) mutant are from (44). Three biological replicates were analyzed for each condition, except for the dehydration 3 h project, which included four biological replicates.

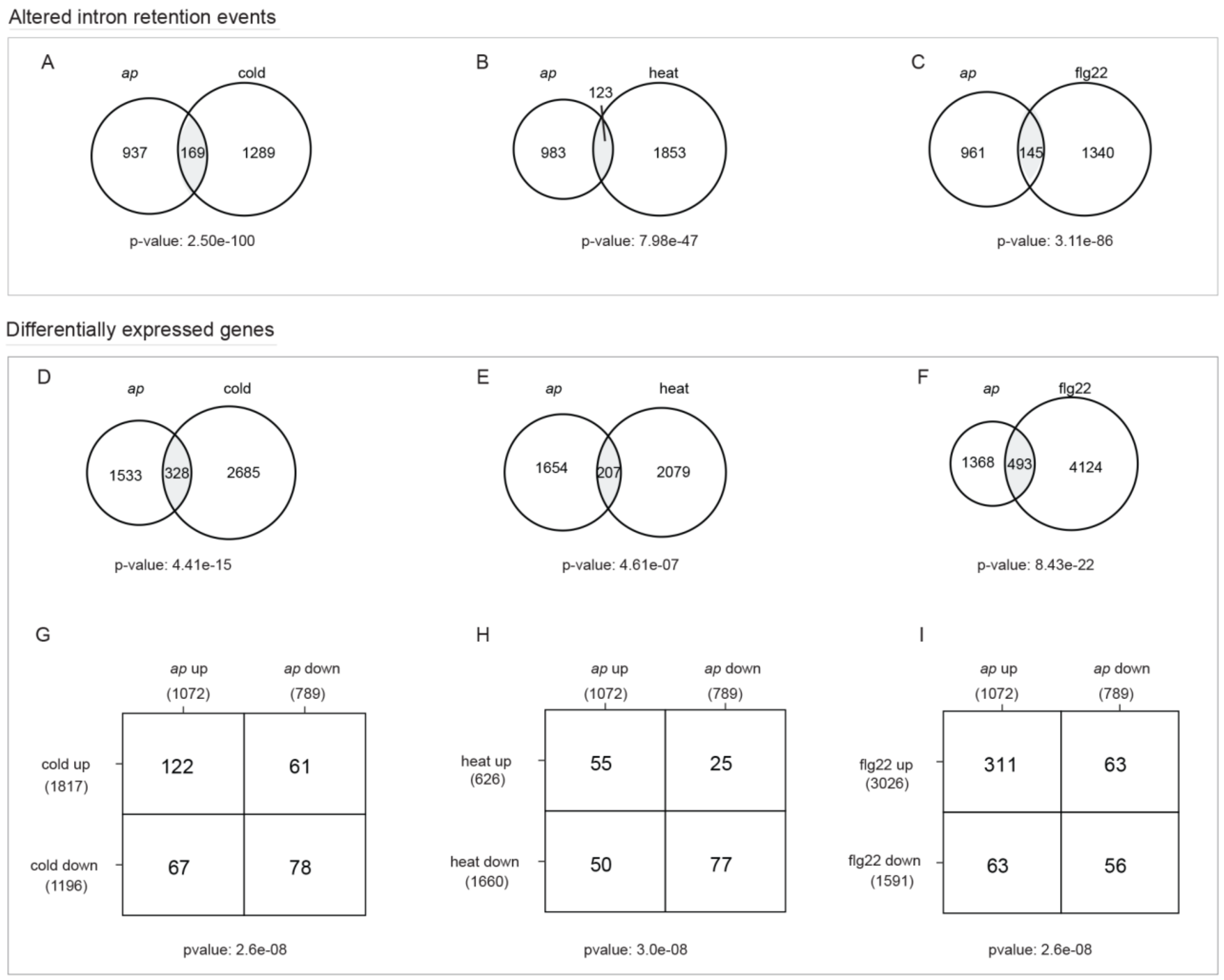

Supplementary Figure S2. Overlap of altered intron retention (IR) events and differentially expressed genes (DEGs) in wild-type upon stress treatment and in the *acinus pinin* mutants.

(**A**–**C**) Altered IR events overlap in wild-type with cold (A), heat (B), and flg22 (C) treatments and in *ap* mutants.

(**D**–**F**) DEGs overlap in wild-type with cold (D), heat (E), and flg22 (F) treatments and in *ap* mutants.

(**G**–**I**) Directional overlap of up- and down-regulated differentially expressed transcripts with cold (G), heat (H), and flg22 (I), classified as concordant (same direction) or discordant (opposite direction).

Statistical significance was assessed using the hypergeometric test (A–F) and Fisher's exact test (G–I).

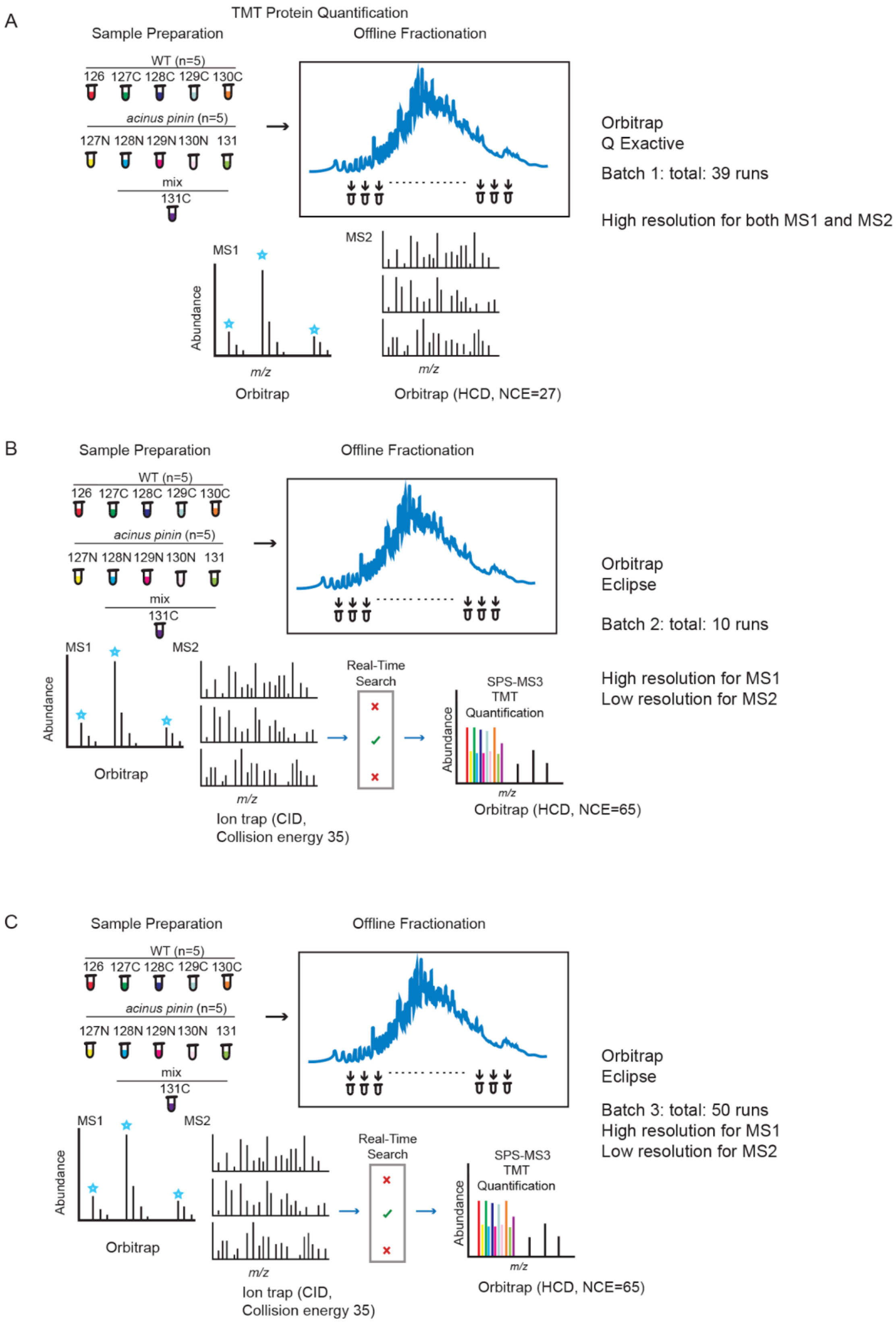
A
TMT Protein Quantification
Sample Preparation
Offline Fractionation
WT (n=5)
126 127C 128C 129C 130C
acinus pinin (n=5)
127N 128N 129N 130N 131
mix
131C
Orbitrap
Q Exactive
Batch 1: total: 39 runs
High resolution for both MS1 and MS2
MS1
MS2
Abundance
m/z
Orbitrap
Orbitrap (HCD, NCE=27)
B
Sample Preparation
Offline Fractionation
WT (n=5)
126 127C 128C 129C 130C
acinus pinin (n=5)
127N 128N 129N 130N 131
mix
131C
Orbitrap
Eclipse
Batch 2: total: 10 runs
MS1
MS2
Abundance
Orbitrap
Real-Time Search
SPS-MS3 TMT Quantification
m/z
Ion trap (CID, Collision energy 35)
Orbitrap (HCD, NCE=65)
High resolution for MS1
Low resolution for MS2
C
Sample Preparation
Offline Fractionation
WT (n=5)
126 127C 128C 129C 130C
acinus pinin (n=5)
127N 128N 129N 130N 131
mix
131C
Orbitrap
Eclipse
Batch 3: total: 50 runs
High resolution for MS1
Low resolution for MS2
MS1
MS2
Abundance
Orbitrap
Real-Time Search
SPS-MS3 TMT Quantification
m/z
Ion trap (CID, Collision energy 35)
Orbitrap (HCD, NCE=65)

Supplementary Figure S3. Tandem Mass Tag (TMT) quantitative analysis of wild-type and *acinus pinin* mutants.

(**A**-**C**) Three batches of TMT experiments were performed: batch 1 on a Q Exactive with MS2-level quantification (panel A), and batches 2 and 3 on an Orbitrap Eclipse with MS3-level quantification (panels B and C). The two Orbitrap Eclipse experiments differ in fractionation numbers. Proteins from WT (5 replicates) and *acinus pinin* mutant (5 replicates) were trypsin-digested and labeled using TMT11-plex. The last channel was assigned to a pooled reference. The labeled peptides were combined and fractionated using high pH reverse-phase HPLC before being analyzed by LC-MS/MS. For the Orbitrap Eclipse, a narrow 0.7 Da precursor isolation window and a real-time library search (RTS)-SPS-MS3 were applied to enhance sensitivity and precision by targeting library-matched TMT peptides and synchronously isolating MS2 fragments for MS3 fragmentation (73).

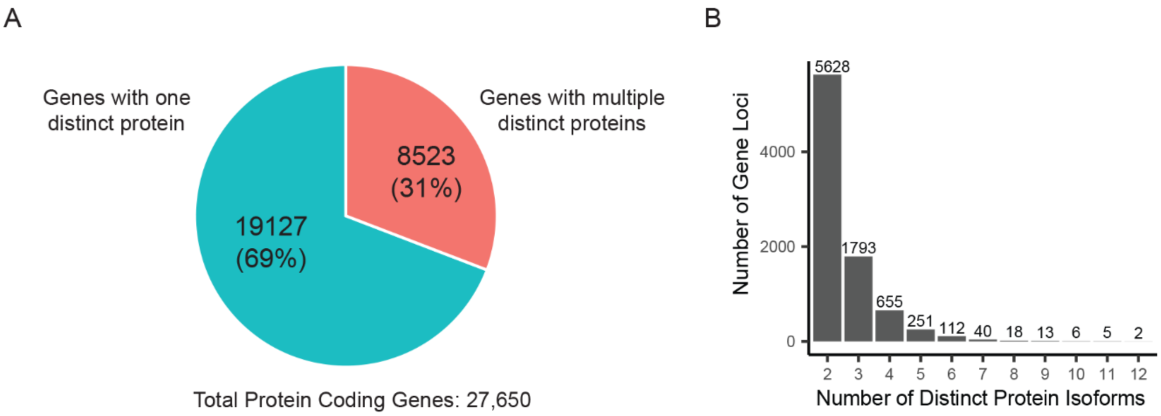

Supplementary Figure S4. Prevalence and distribution of alternative isoforms in the Araport11 annotation.

(A) Proportion of genes predicted to produce a single isoform versus multiple isoforms. Approximately 31% of genes are annotated with more than one isoform protein. Genes that have different transcript isoforms but produce only one unique protein or similar protein sequence are collapsed.

(B) Distribution of the number of protein isoforms among genes with multiple isoforms in Araport11 database.

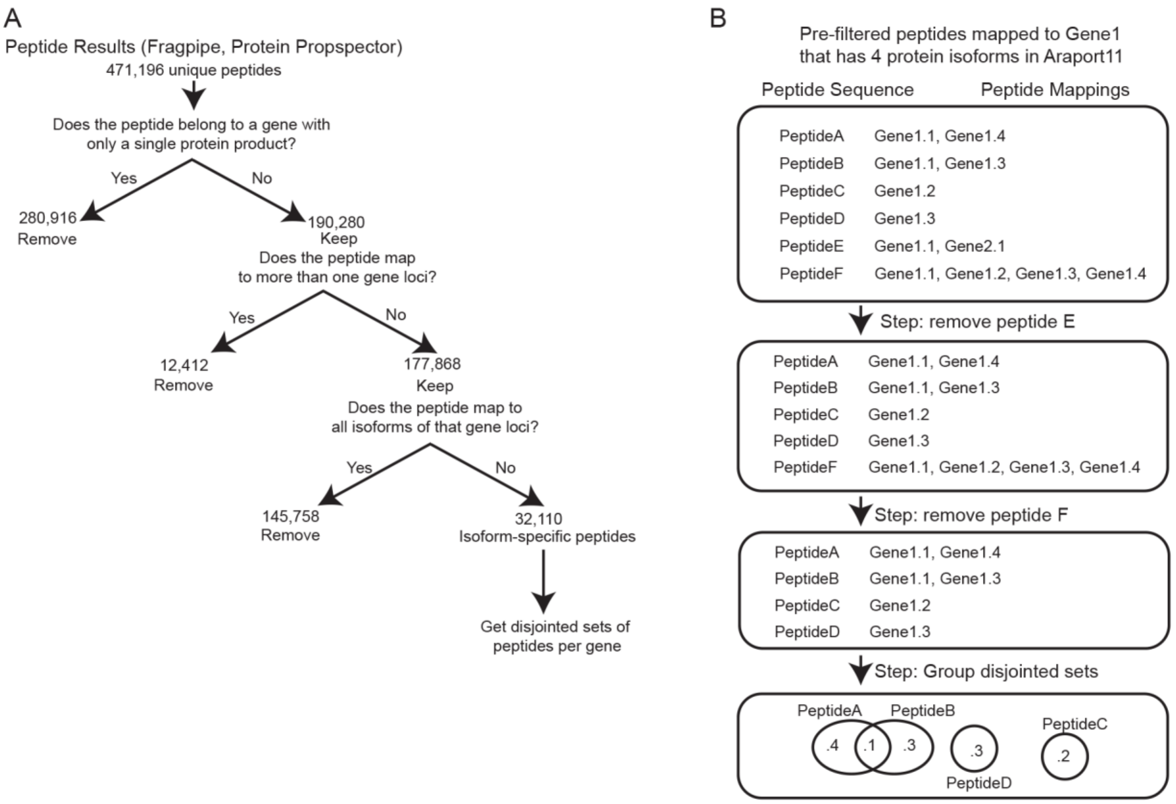

Supplementary Figure S5. Workflow for protein isoform calculation.

(**A**) Summary of the filtering strategy. From an initial pool of 471,196 identified peptides, a multi-step filtering process was employed to isolate isoform-specific sequences. First, 280,916 peptides associated with single-isoform protein products were excluded, followed by the removal of 12,412 peptides that mapped to multiple gene loci. Finally, 145,758 common peptides were discarded; these represent sequences shared among all isoforms of their corresponding multi-isoform gene loci. This subtraction pipeline yielded a final set of 32,110 isoform-specific peptides for downstream isoform mapping.

(**B**) Representative schematic of peptide exclusion and isoform mapping logic. This simplified model illustrates the filtering of 190,280 peptides using a hypothetical Gene 1 with four potential isoforms (.1–.4). Six detected peptides (A–F) are mapped to support the exclusion criteria: Peptide E is removed for mapping to multiple gene loci, while Peptide F is excluded as a constitutive sequence shared across all isoforms. Among the remainder, Peptide A maps to isoforms .1/.4, B to .1/.3, C uniquely to .2, and D uniquely to .3. Based on the principle of parsimony, the minimum set of isoforms required to explain these detections consists of three isoforms: .2, .3, and either .1 or .4. While this imaginary case uses a minimal number of peptides for clarity, most experimental isoform assignments in this study are supported by a more extensive density of peptide evidence.

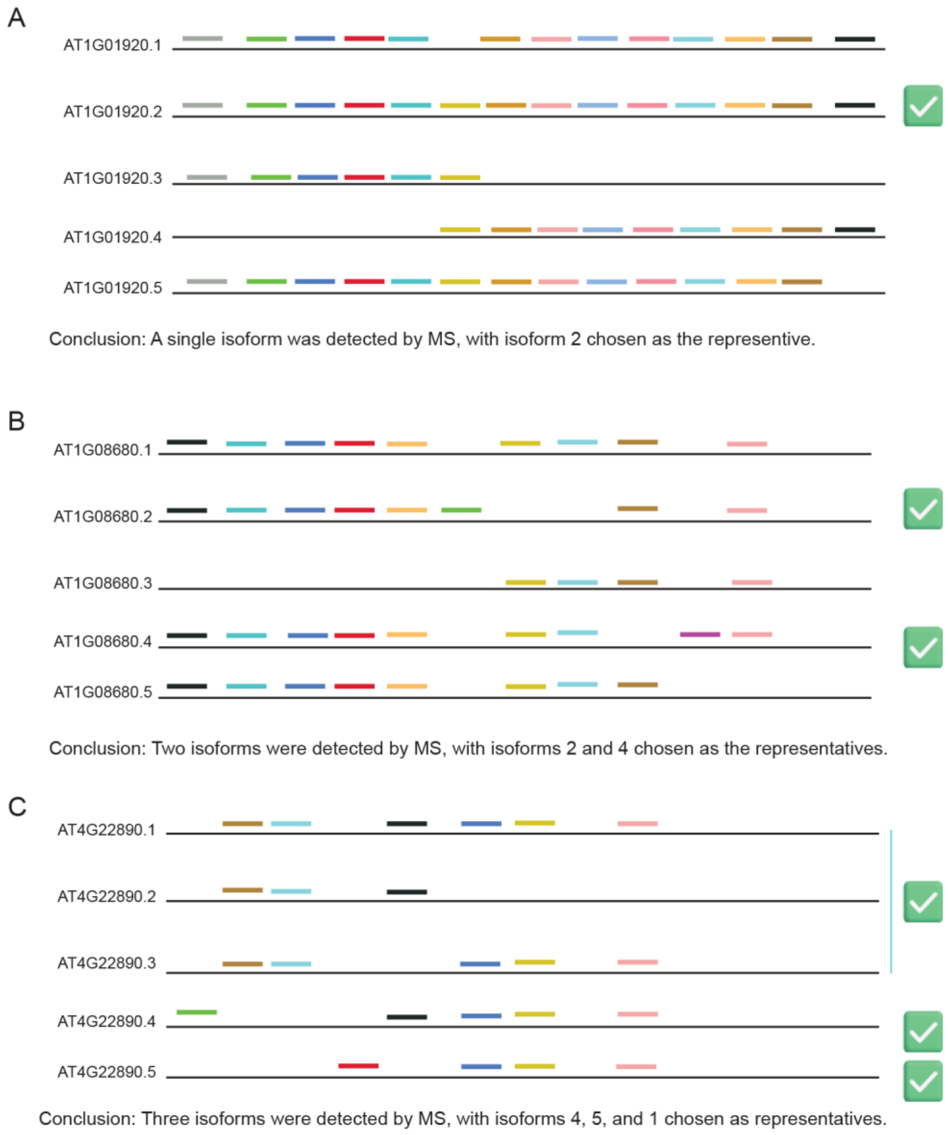

Supplementary Figure S6. Peptide-based isoform analysis for genes with multiple annotated isoforms.
(**A**–**C**) Schematics illustrating peptide assignments and isoform quantification for AT1G01920, AT1G08680, and AT4G22890. Calculations are based on identifying the most parsimonious set of protein isoforms required to explain the observed peptide evidence; for *AT1G01920*, the minimum set required is the .2 isoform, while *AT1G08680* requires two (.2 and .4), and *AT4G22890* requires three (.4, .5, and the .1/.2/.3 group). This approach provides a conservative estimate of the proteoform landscape, the complexity of which may be higher and further resolvable with the support of full-length RNA sequencing. Colored lines represent unique peptides aligned to each predicted isoform.

Step 1: Convert Strings to Sets

Let $S = \{s_1, s_2, \ldots, s_n\}$ be the set of input strings. Each string s_i represents a numeric set that corresponds to isoforms matched by a given peptide:

$$A_i = \{a_{i1}, a_{i2}, \ldots, a_{im_i}\}, \quad A_i \subseteq \mathbb{N}$$

Define the collection of sets as:

$$\mathcal{A} = \{A_1, A_2, \ldots, A_n\}$$

Step 2: Filter Sets with Disjointness Condition

We retain only those sets that are disjoint from at least one other:

$$\mathcal{A}' = \{A_i \in \mathcal{A} \,|\, \exists j \neq i \text{ such that } A_i \cap A_j = \emptyset\}$$

If $\mathcal{A}' = \emptyset$, return the empty list.

Step 3: Group Directly Overlapping Sets

Let $\mathcal{N} = \{n_1, n_2, \ldots, n_k\}$ be identifiers (e.g., original strings) for the sets in $\mathcal{A}'$. For each $A_i \in \mathcal{A}'$, define a group G_r such that:

$$G_r = \{n_j \in \mathcal{N} \,|\, A_i \cap A_j \neq \emptyset \text{ and } i \neq j\} \cup \{n_i\}$$

Note: Overlaps are considered only directly (non-transitive).

Step 4: Enforce Singleton Restriction

For each group G_r, define:

$$S_r = \{n_i \in G_r \mid |A_i| = 1\}, \quad \text{(singleton sets)}$$
$$O_r = G_r \setminus S_r \quad \text{(non-singleton sets)}$$

Then:

- If $|S_r| \leq 1$, keep the group as:

$$G'_r = O_r \cup S_r$$

- If $|S_r| > 1$, construct:

$$G'_r = O_r \cup \{s_{r1}\}, \quad \text{(keep one singleton)}$$
$$\text{and } \{s_{r2}\}, \{s_{r3}\}, \ldots \quad \text{(each remaining singleton in its own group)}$$

Supplementary Figure S7. Workflow for filtering and grouping numeric sets.

Numeric sets represent isoforms mapped by each peptide. (1) Convert peptide mappings into numeric sets. (2) Retain sets disjoint from at least one other set. (3) Group sets with direct overlaps. (4) Retain only one singleton per group; additional singletons are reassigned to separate groups. This figure was generated by AI based on the R code used in our isoform calculation algorithm.

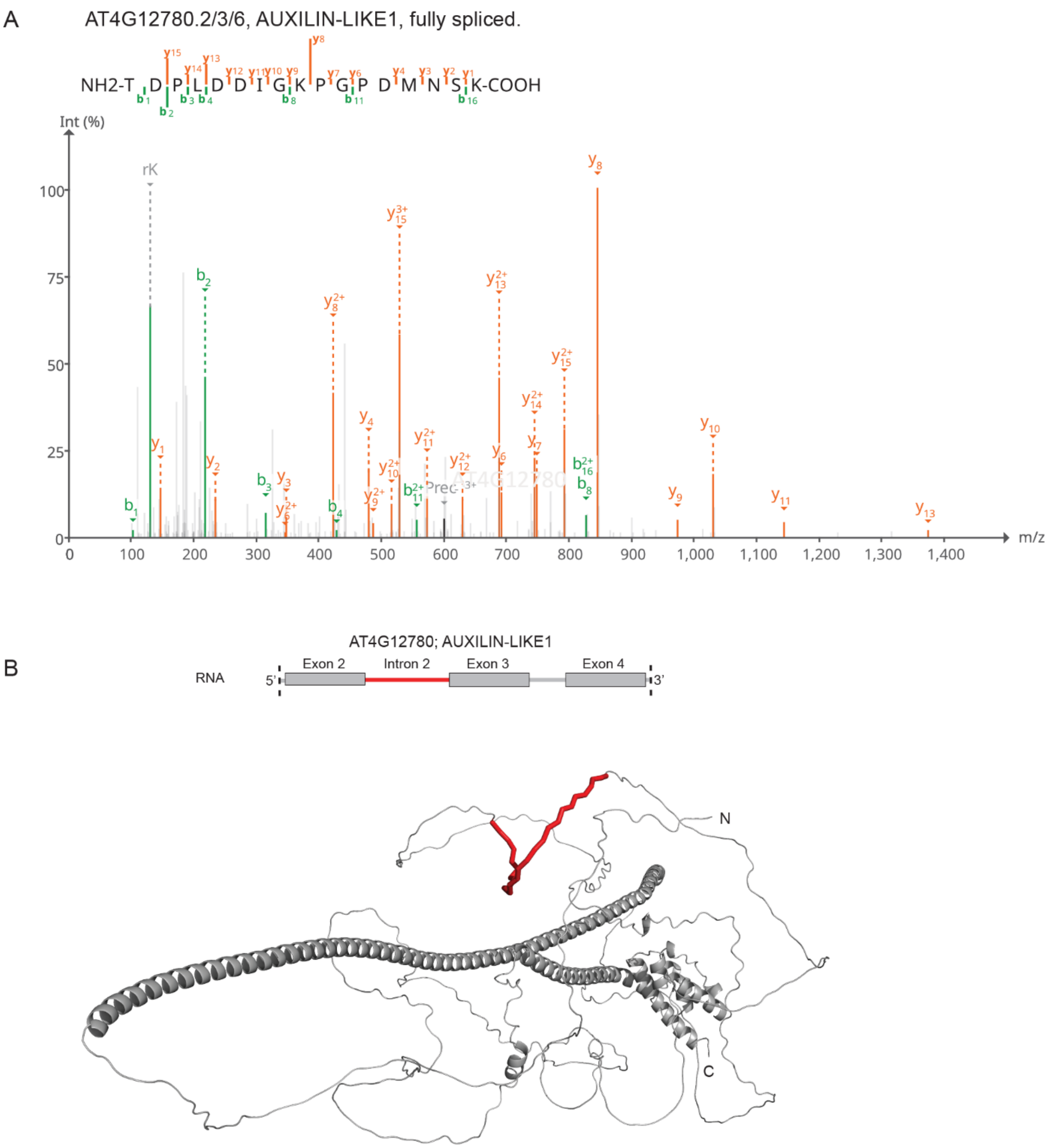

Supplementary Figure S8. Spectrum of a junction peptide supporting the spliced form of AT4G12780, and the AlphaFold-predicted structure illustrating the location of the additional sequence resulting from intron retention.

(A) MS2 spectrum of a peptide spanning the junction between exons 2 and 3 of *AT4G12780.2/3/6*, supporting the fully spliced Auxilin-like 1 isoform. The spectrum exhibits extensive b- and y-ion coverage, unambiguously identifying exon 2–3 junction peptide sequence.

(B) AlphaFold-predicted structure of AT4G12780, in which the retained intronic sequence is highlighted in red. The additional 27 amino acids derived from intron retention are incorporated into a predicted unstructured region based on AlphaFold model ID: AF-Q9SU08-F1.

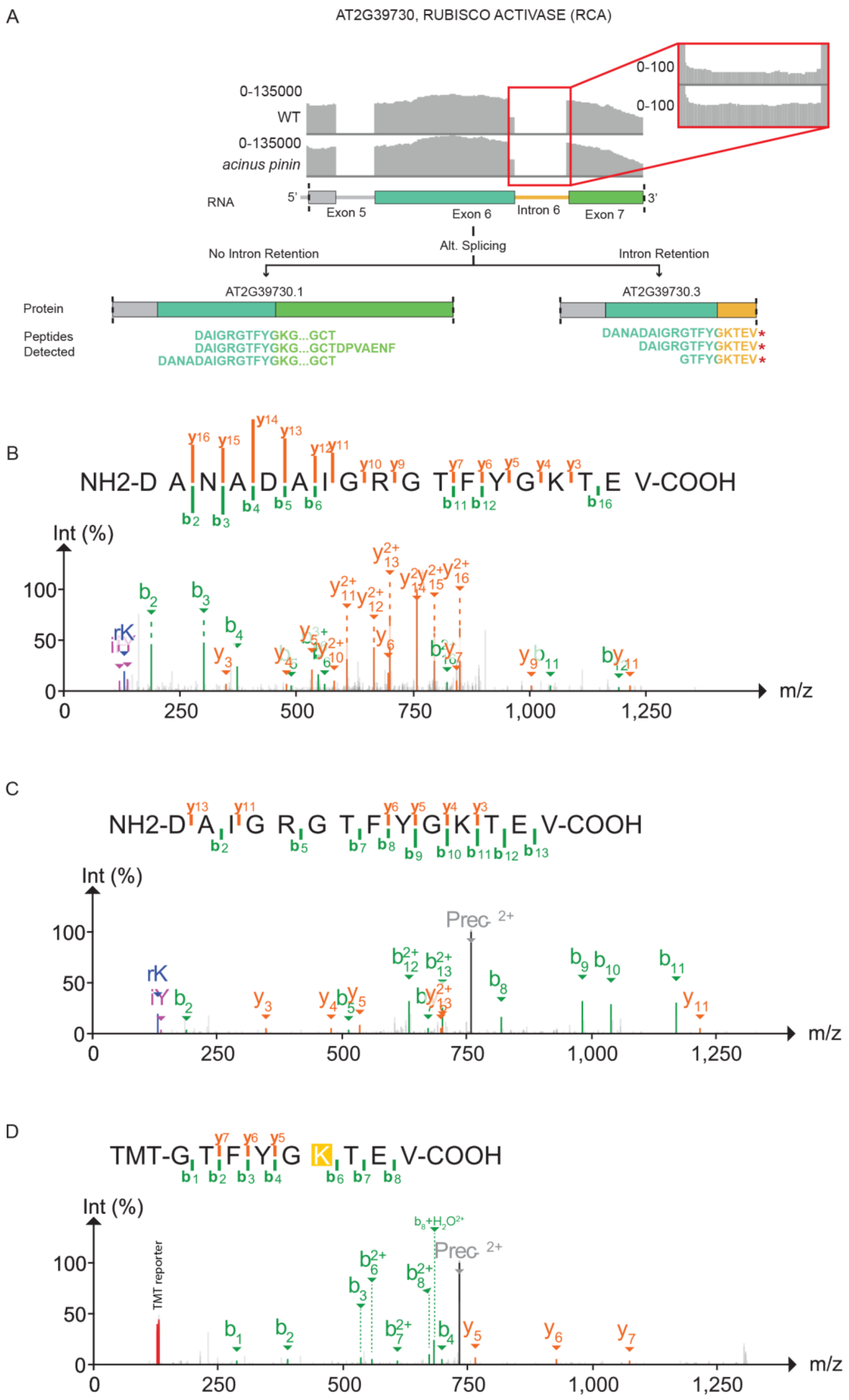

A
AT2G39730, RUBISCO ACTIVASE (RCA)
0-135000
WT
0-135000
acinus pinin
0-100
0-100
RNA
Exon 5
Exon 6
Intron 6
Exon 7
Alt. Splicing
No Intron Retention
Intron Retention
AT2G39730.1
AT2G39730.3
Protein
Peptides Detected
DAIGRGTFYGKG...GCT
DAIGRGTFYGKG...GCTDPVAENF
DANADAIGRGTFYGKG...GCT
DANADAIGRGTFYGKTEV*
DAIGRGTFYGKTEV*
GTFYGKTEV*
B
NH2-D A N A D A I G R G T F Y G K T E V-COOH
Int (%)
m/z
C
NH2-D A I G R G T F Y G K T E V-COOH
Int (%)
Prec 2+
m/z
D
TMT-G T F Y G K T E V-COOH
Int (%)
TMT reporter
Prec 2+
m/z

Supplementary Figure. S9. RNA-seq and mass spectra evidence supporting the AT2G39730.3 intron-retained isoform.
(**A**) Genomic visualization of RNA-seq reads showing the retention of intron 6 in both WT and *acinus pinin* mutants.
(**B–C**) Annotated mass spectra of two peptides specific to the AT2G39730.3 isoform. Both peptides were generated via AspN digestion and identified in both WT and *acinus pinin* mutant samples.
(**D**) Annotated mass spectrum of a TMT-labeled peptide generated via Trypsin digestion, supporting the AT2G39730.3 annotation. Both the N-terminus and the Lysine (K) residue are labeled with the TMT tag.

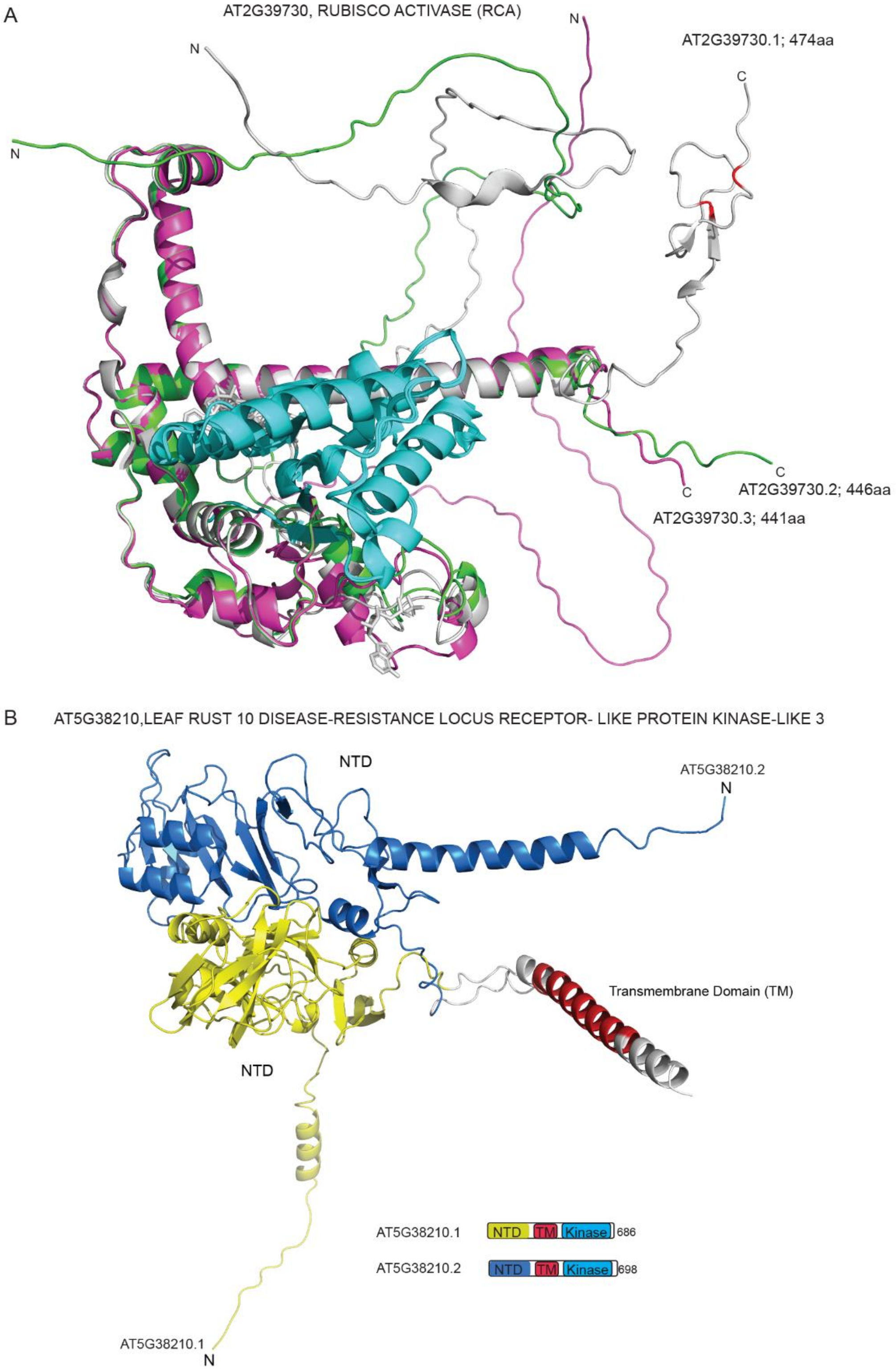

Supplementary Figure S10. Structure information for protein isoforms of RCA and LRK10L3.

(A) AlphaFold-predicted structures of three RCA isoforms (UniProt ID: P10896) showing a shared N-terminal region but variable C-terminal domains. The full-length isoforms contain two C-terminal cysteine residues highlighted in red, which are implicated in redox regulation.

(B) AlphaFold-predicted structures of two LRK10L3 isoforms (AlphaFold model IDs: AF-A0A1P8BDK9-F1 and AF-Q8VYG0-F1) showing distinct N-terminal regions and a shared transmembrane (TM) domain. The C-terminal kinase domain is omitted for clarity.

A AT5G20920, EIF2 BETA

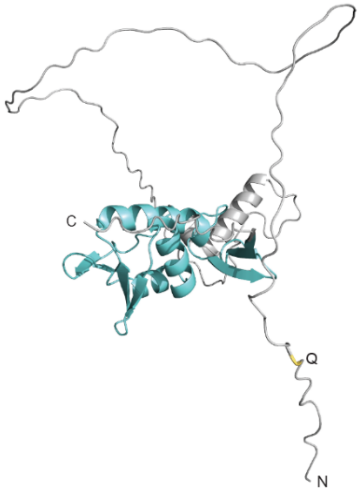

B AT2G27720, RIBOSOMAL PROTEIN P2Z

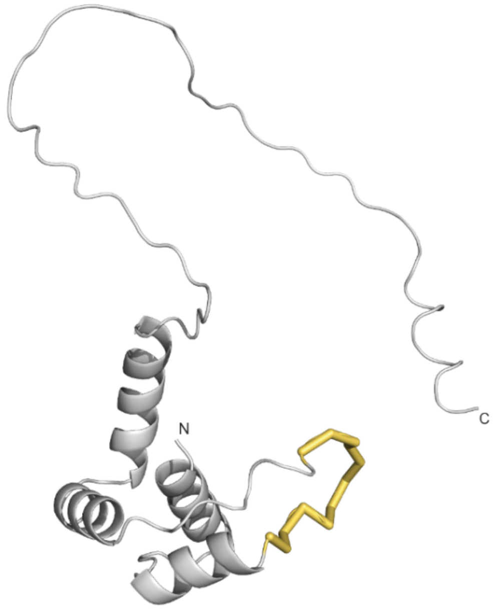

Supplementary Figure S11. Structure information for EIF2 BETA and RIBOSOMAL PROTEIN P2Z.

(**A**) AlphaFold-predicted structure of EIF2 BETA (AF-Q41969-F1-model_v6), with the glutamine (Q) residue added by alternative splicing highlighted.

(**B**) AlphaFold-predicted structures of ribosomal protein P2Z (AlphaFold model IDs: AF-A0A1P8BDK9-F1 and AF-Q8VYG0-F1), showing the additional 12-amino-acid sequence difference (no exon3 skipping) highlighted in yellow.

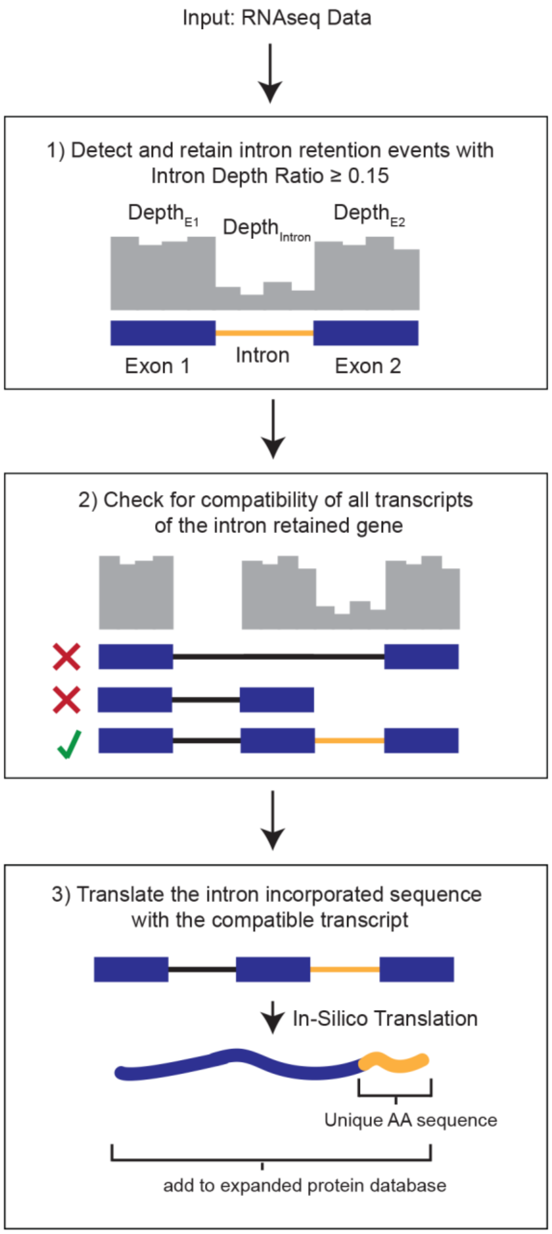

Supplementary Figure S12. Workflow for generating a custom peptide database from transcripts containing unannotated retained introns.

This multi-step pipeline generates novel peptide sequences from transcripts with retained introns identified via RNA-seq. (1) Intron retention events are filtered to exclude low-confidence cases (Intron Depth Ratio < 0.15). (2) Retentions compatible with annotated exon boundaries in Araport11 are retained (i.e., flanked by two exons within the same transcript). Because RackJ collapses transcripts by gene loci before defining introns, intron boundaries defined by RackJ are not always directly compatible with the original transcript structure. Step 2 ensures that each intron is correctly integrated into its transcript. (3) Retained introns are incorporated into modified transcripts, translated *in silico* to produce protein sequences, and appended to the Araport11 proteome for downstream database searches.

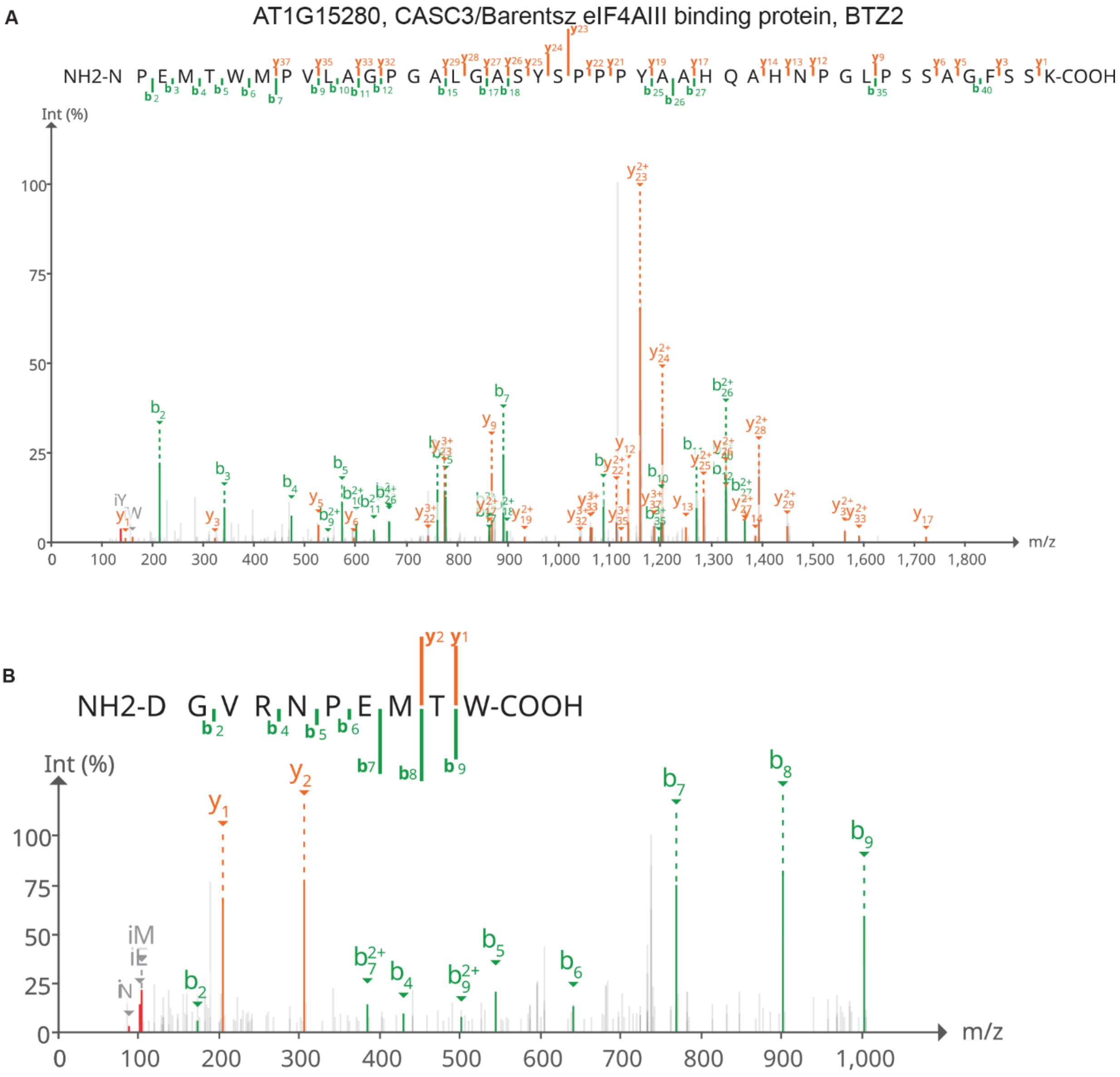

Supplementary Figure S13. MS2 spectra of peptides supporting both the spliced and intron-retained isoforms of AT1G15280 (BTZ2); the intron-retained isoform is not annotated in Araport11.

(A) Peptide spanning the exon 6–exon 7 junction of the spliced isoform AT1G15280.1.
(B) Peptide spanning the exon 6–intron 6 junction of the intron-retained isoform. The peptide was detected in both WT and *acinus pinin* mutants.

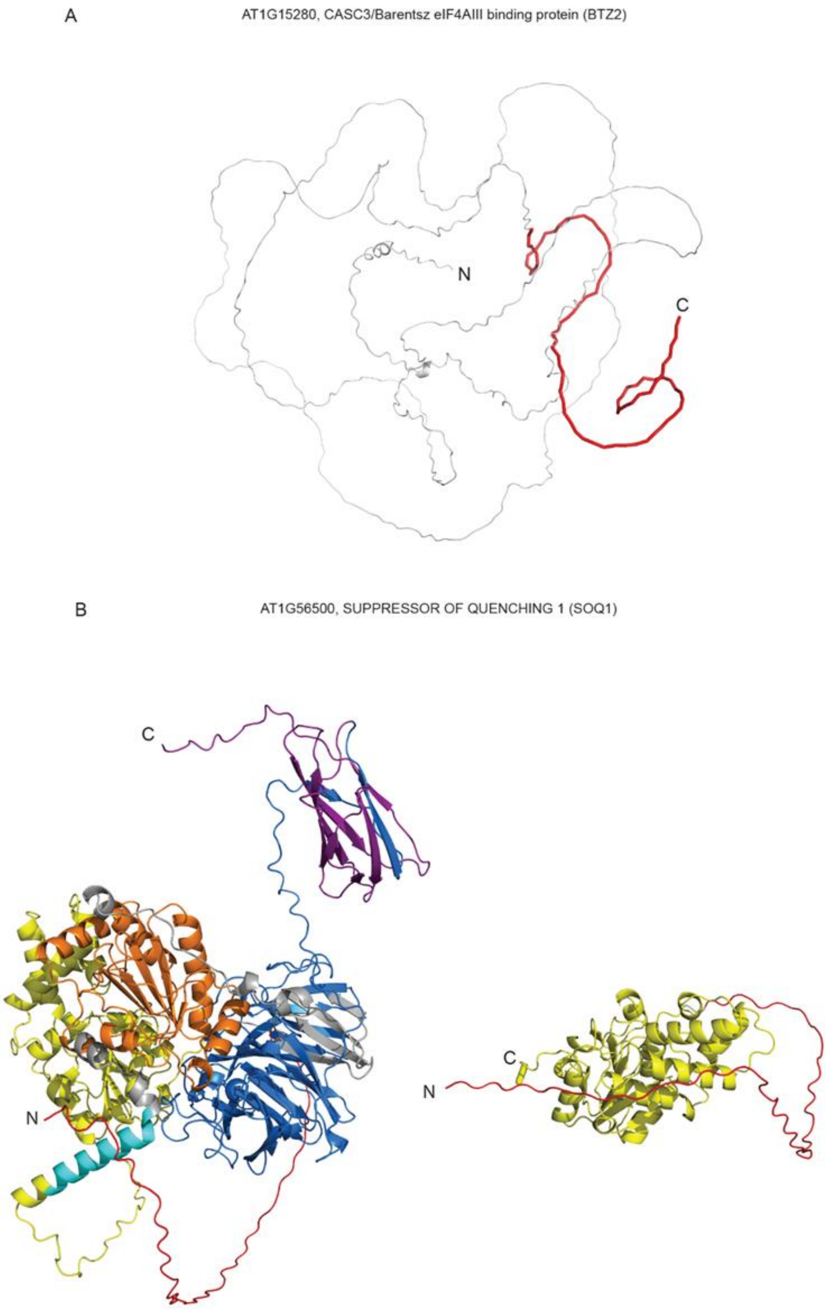

Supplementary Figure S14. Structural information for BTZ2 and SOQ1.
(**A**) AlphaFold-predicted structure of BTZ2, showing that the protein is largely disordered. Intron retention of intron 6 introduces a premature stop codon, resulting in a truncated protein that is 84 amino acids shorter. The missing region is highlighted in red.

(**B**) Structural comparison of the full-length (left) and truncated (right) forms of SOQ1. The color scheme is consistent with that in Figure 4F.

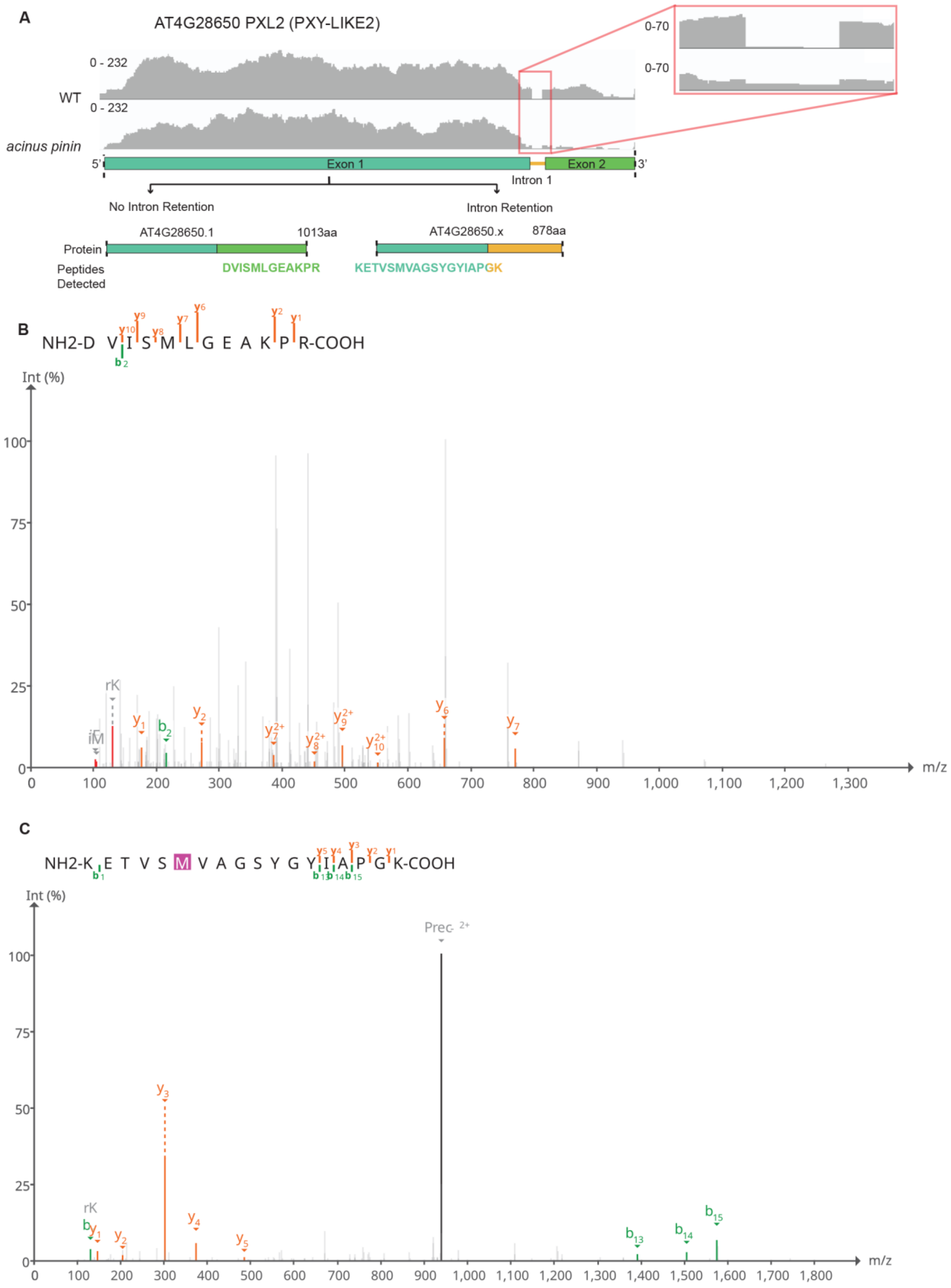
A
AT4G28650 PXL2 (PXY-LIKE2)
0 - 232
WT
0 - 232
acinus pinin
0-70
0-70
5'
Exon 1
Exon 2
3'
Intron 1
No Intron Retention
Intron Retention
AT4G28650.1
1013aa
AT4G28650.x
878aa
Protein
Peptides Detected
DVISMLGEAKPR
KETVSMVAGSYGYIAPGK
B
NH2-D V I S M L G E A K P R-COOH
Int (%)
m/z
C
NH2-K E T V S M V A G S Y G Y I A P G K-COOH
Int (%)
Prec 2+
m/z

Supplementary Figure S15. Proteomic evidence for two isoforms of AT4G28650 (PXY-LIKE 2): the annotated spliced form and an intron-retained isoform absent from Araport11.
(**A**) RNA-seq coverage and gene annotation showing retention of intron 1 in both WT and *acinus pinin*. Inset highlights reads spanning intron 1, revealing increased retention in the *acinus pinin* mutant.
(**B**) MS2 spectrum detecting the peptide encoded by exon 2, supporting the canonical spliced isoform (AT4G28650.1).
(**C**) MS2 spectrum detecting the junction peptide spanning exon 1 and intron 1, supporting a previously unannotated intron-retained isoform (AT4G28650.x). Retention of intron 1 introduces a stop codon, resulting in a truncated protein of 878 amino acids. This junction peptide was detected in multiple tissues across cell atlas datasets.

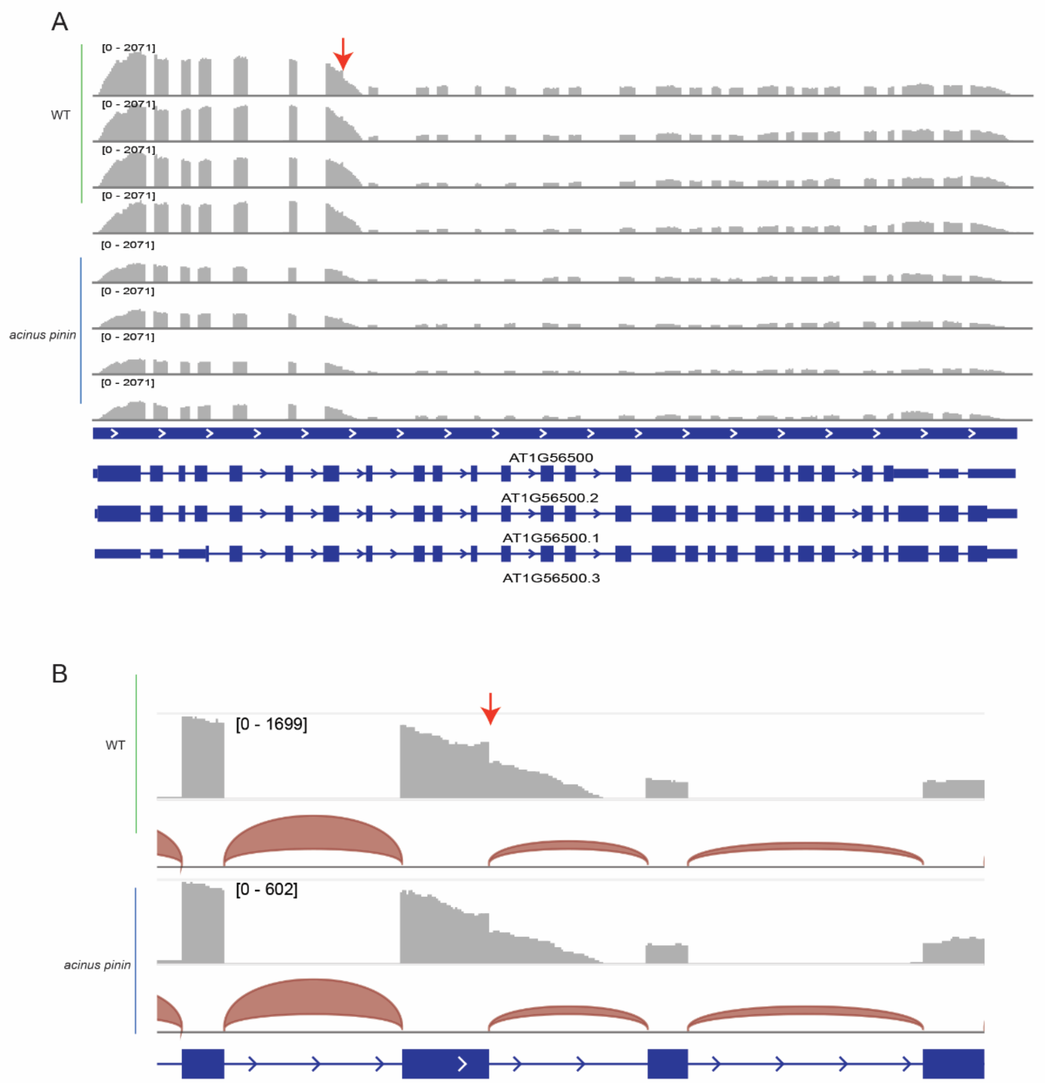

Supplementary Figure S16. RNA-seq coverage indicates the need for a revised gene model of AT1G56500.

(**A**) RNA-seq results and schematic annotation of canonical AT1G56500 isoforms are shown. Coverage from four biological replicates of WT and *acinus pinin* shows consistent retention of intron 7, accompanied by a sharp drop in downstream reads. This pattern suggests that, in addition to the current annotation (AT1G56500.1/2/3), a shorter mRNA isoform is generated via intron 7 retention and alternative 3’ processing within intron 7, with transcript levels reduced in the *acinus pinin* mutant.

(**B**) Detailed Sashimi plot illustrating the splicing junctions and transcript architecture for isoforms AT1G56500.1/2/3 and the novel AT1G56500.x variant. The plot highlights the junction read support for both the canonical splicing events and the specific intron retention/alternative polyadenylation events described.

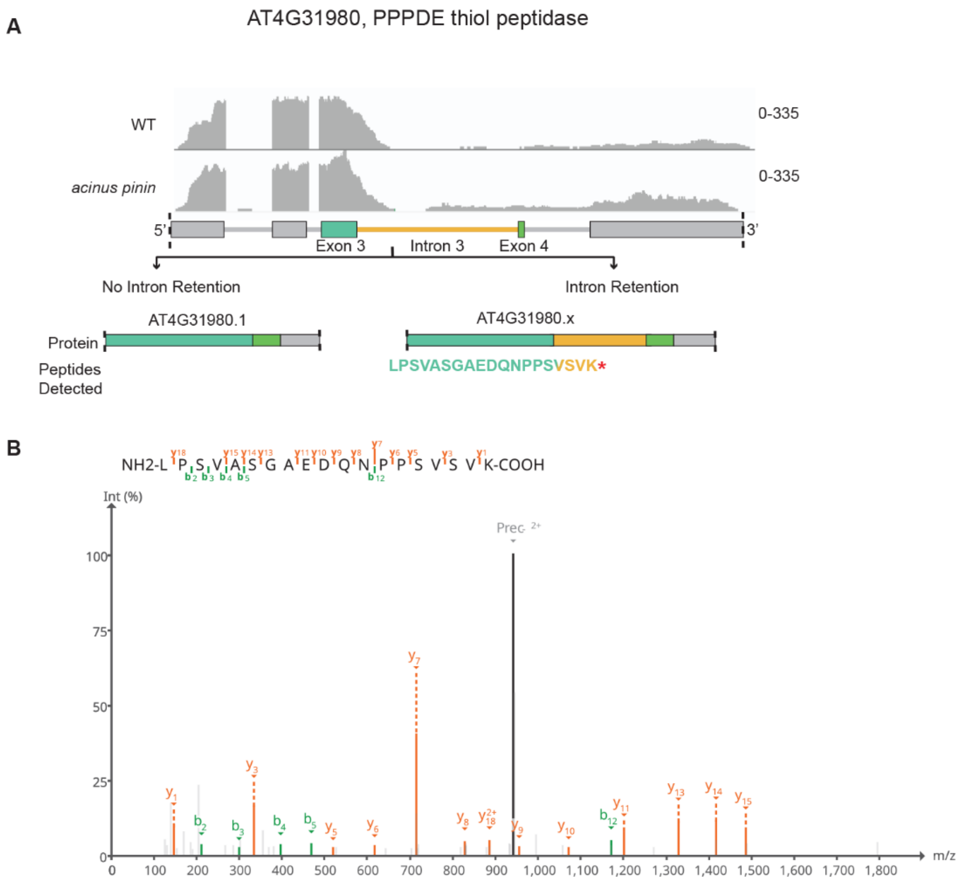

Supplementary Figure S17. Proteomic and transcriptomic analyses reveal a new isoform of AT4G31980 (PPPDE thiol peptidase), which includes intron 3.

(**A**) RNA-seq coverage is compared with the Araport11 annotation. The reads reveals robust coverage across exons 1–3 and the 5′ region of intron 3. There is a gradual decline in read coverage across the remainer of the intron and downstream regions. This suggests that the current gene model requires refinement. However, expression of the annotated isoform AT4G31980.1 cannot be ruled out based on the 3' reads of the gene. Several AT4G31980 peptides were identified; however, peptides supporting the junction of exons 3 and 4 were not detected.

(**B**) The MS2 spectrum identifies a peptide that spans exon 3 and intron 3. The extensive b- and y-ion series support unambiguous identification of the peptide.

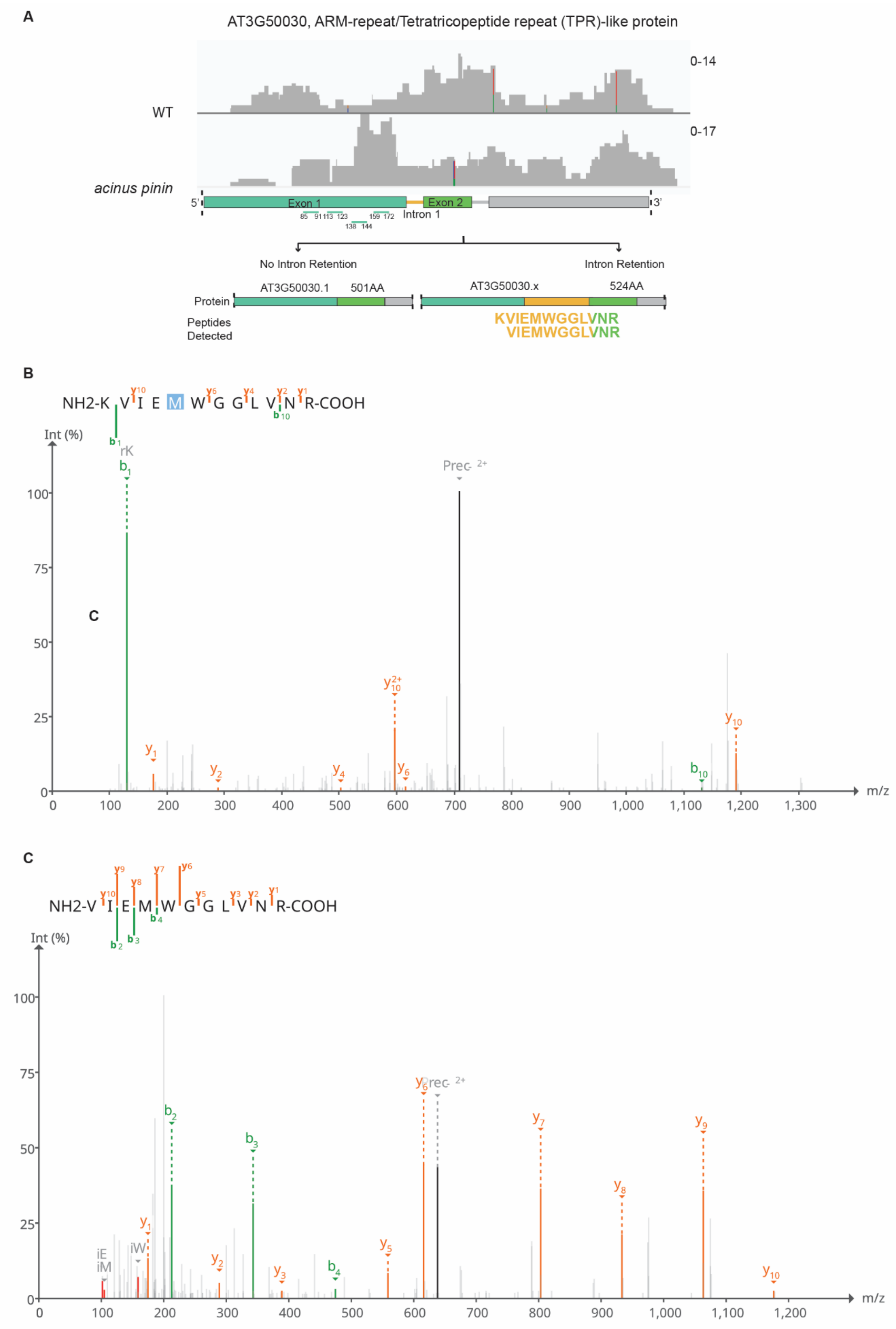
A
AT3G50030, ARM-repeat/Tetratricopeptide repeat (TPR)-like protein
WT
0-14
acinus pinin
0-17
5'
Exon 1
Exon 2
3'
Intron 1
No Intron Retention
Intron Retention
Protein
AT3G50030.1
501AA
AT3G50030.x
524AA
Peptides Detected
KVIEMWGGLVNR
VIEMWGGLVNR
B
NH2-K V I E M W G G L V N R-COOH
Int (%)
Prec 2+
m/z
C
NH2-V I E M W G G L V N R-COOH
Int (%)
Prec 2+
m/z

Supplementary Figure S18. Proteomic evidence supports translation of annotated intron 2 in AT3G50030 (ARM-like/tetratricopeptide repeat protein), suggesting a revised isoform.
(**A**) RNA-seq coverage and gene annotations from Araport11 (AT3G50030.1) and the proposed revised model (AT3G50030.x). The revised model includes intron 1, although low RNA-seq coverage makes precise boundary determination challenging. Intron 1 is in-frame and lacks a stop codon.
(**B**–**C**) Two peptides were identified that map to intron 1, providing evidence for its translation and suggesting that the current Araport11 annotation may require revision. Although TAIR currently annotates AT3G50030 as “not expressed in wild-type plants,” our proteomic analysis detected multiple unique peptides corresponding to this protein. In contrast, no splice-junction peptides were identified to support the annotated isoform AT3G50030.1. In addition, four peptides mapping to exon 1 were detected, spanning amino acid residues 85–91, 113–123, 138–143, and 159–172.

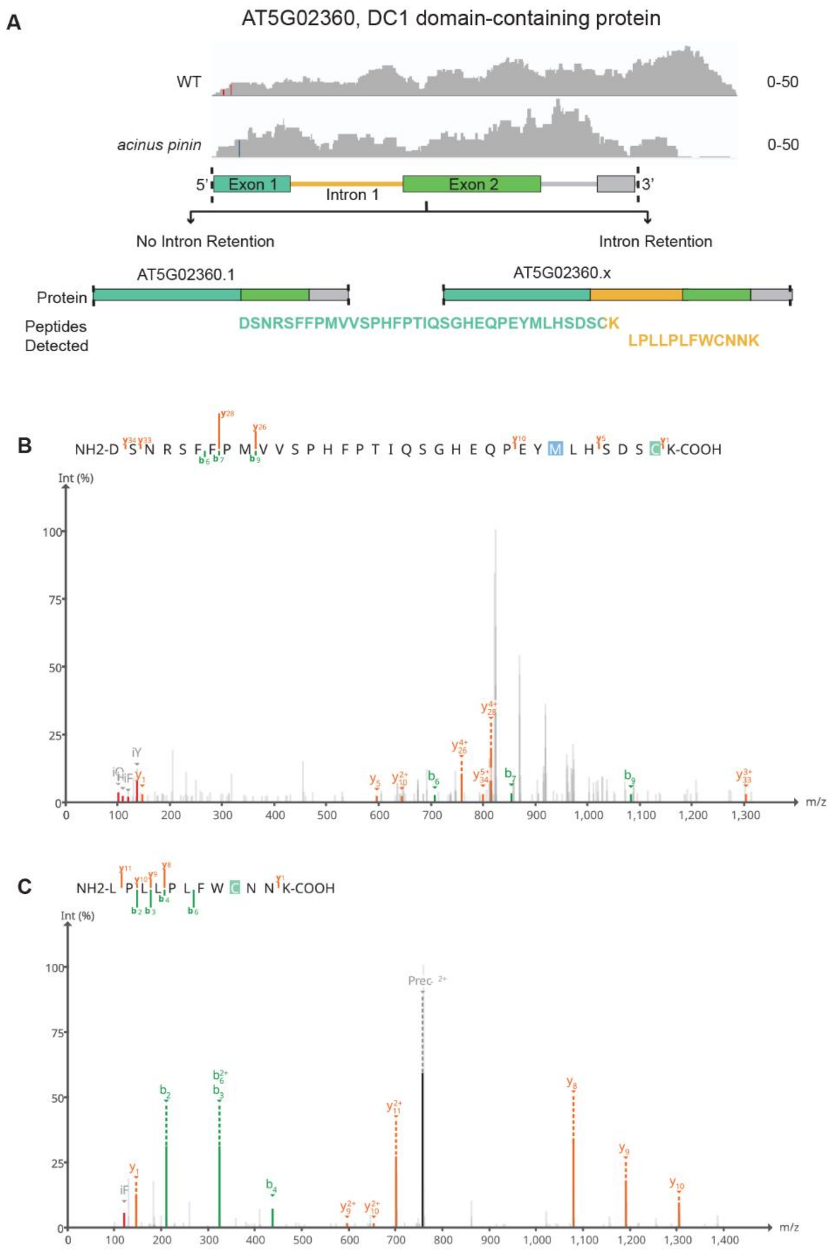

Supplementary Figure S19. Proteomic evidence supports a revised annotation of AT5G02360 (DC1 domain-containing protein) compared with the current annotation (AT5G02360.1).

(**A**) RNA-seq coverage and Araport11 annotation for AT5G02360. Although RackJ interprets these reads as intron retention based on the existing annotation, the low RNA-seq coverage makes precise definition of intron boundaries difficult. JBrowse (arabidopsis.org) shows some junction reads supporting splicing of AT5G02360.1.

(**B**-**C**) Two independent MS2 spectra provide peptide-level evidence supporting a revised annotation in which intron 1 is retained and translated. Multiple peptides were detected for AT5G02360, but no isoform-specific peptides were identified for AT5G02360.1.

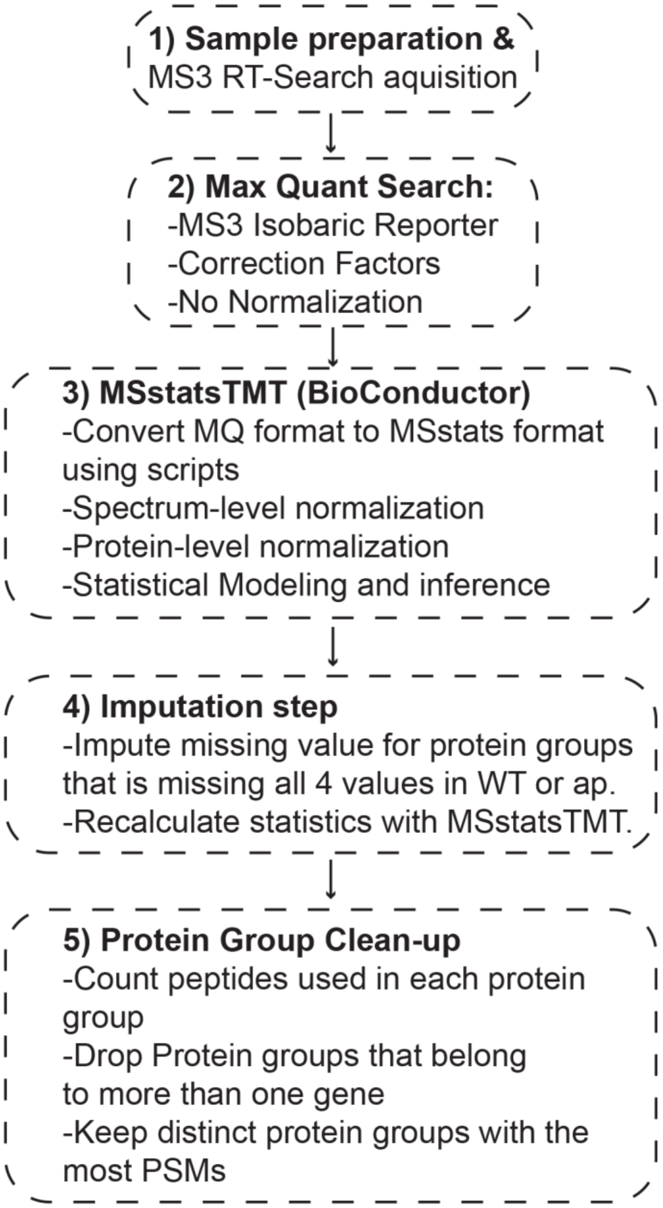

Supplementary Figure S20: Workflow for protein-level quantification using MS3 real-time library search data.

Raw MS3 data were processed in MaxQuant with the search type set to MS3 Isobaric Reporter. Correction factors were applied as appropriate, and normalization within MaxQuant was omitted. MSstatsTMT was then used for normalization and statistical analysis. To generate a single quantification value per protein, protein groups were filtered to retain only those uniquely mapping to a single gene. When multiple protein groups corresponded to the same gene, the group with the highest number of detected peptides was selected.

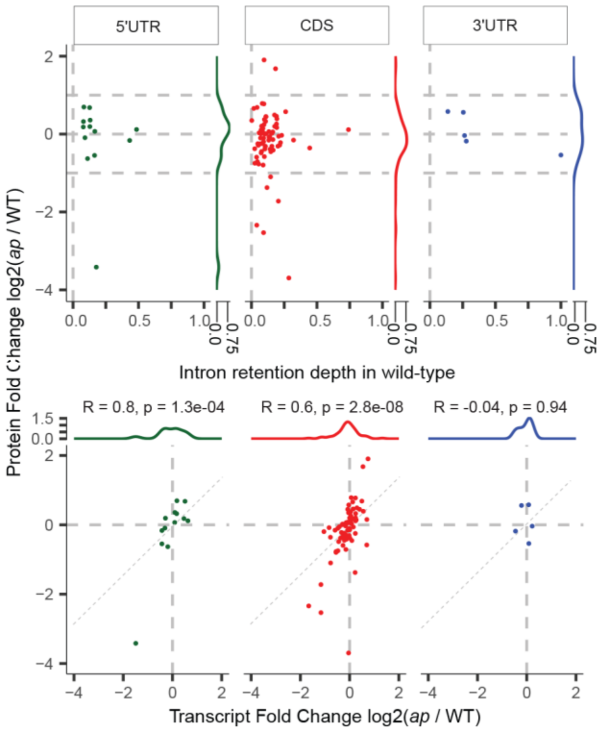

Supplementary Figure S21. Effects of retained introns that are reduced in *acinus pinin* mutants on transcript and protein abundance.

Density plots show distribution changes in protein (top) and RNA (bottom) levels for genes with retained introns that are reduced in *acinus pinin* mutants. Intron retention depth in wild-type is shown in the top panel. Interpretation is limited due to the small number of events and potential confounding effects from increased retention of other introns in the same genes in the mutant. 5’UTR, n=13; CDS, n=74; 3’ UTR, n=5.
